# Leptomeningeal Disease Secondary to Invasive Ductal Carcinoma of the Breast: A Rapidly Progressive and Fatal Complication

**DOI:** 10.7759/cureus.74421

**Published:** 2024-11-25

**Authors:** Saurav Dhawan, Fredwin Mattathil, Isha Malik, Mantripragada Khyathi, Bhawna Bhakar

**Affiliations:** 1 Internal Medicine, Manchester University NHS Foundation Trust, Manchester, GBR; 2 Internal Medicine, North Manchester General Hospital, Manchester, GBR; 3 Diabetes and Endocrinology, North Manchester General Hospital, Manchester, GBR; 4 Internal Medicine, Stockport NHS Foundation Trust, Stockport, GBR; 5 Internal Medicine, Oxford University Hospitals NHS Foundation Trust, Oxford, GBR

**Keywords:** absence seizures, aseptic meningitis, breast cancer metastasis, leptomeningeal disease (lmd), tb meningitis

## Abstract

Leptomeningeal disease (LMD) is a rare yet serious complication of advanced malignancy, often seen in breast cancer and associated with a poor prognosis.

This case report highlights the rapid progression and diagnostic challenges encountered in a woman in her 40s with advanced breast cancer who presented with severe headaches, absence seizures, and diplopia. The patient's complex past history included invasive ductal carcinoma, prior brain metastasis, and recent craniotomy, which added significant challenges to diagnosis and management.

Clinical investigations included computed tomography (CT) of the head, magnetic resonance imaging (MRI) of the orbit and head, electroencephalography (EEG), and lumbar puncture, which indicated optic nerve sheath enhancement, ventricular prominence, coating of facial and vestibulocochlear nerves, and cerebrospinal fluid abnormalities suggestive of LMD. Although empiric treatment for bacterial and tuberculous meningitis was initiated, the patient's rapid decline necessitated an aggressive multimodal approach. Management included high-dose corticosteroids, broad-spectrum antibiotics, antitubercular drugs, and anticonvulsants, though her seizures persisted. Ultimately, comfort-focused care was prioritized as her condition remained refractory to interventions, and she was transitioned to palliative care.

This case emphasizes the need for early suspicion, prompt multidisciplinary involvement, and the challenges in managing complex oncological cases with atypical neurological manifestations. The poor prognosis associated with LMD reflects the limitations of current therapeutic strategies and the need for a nuanced approach to care.

## Introduction

Leptomeningeal disease (LMD) occurs when tumor cells infiltrate the leptomeninges, subarachnoid space, and other compartments within the cerebrospinal fluid (CSF) [1]. It often presents with subtle neurological symptoms which can be difficult to delineate. Imaging may reveal either localized areas of enhancement or a more diffuse pattern. This leads to symptoms resulting from dysfunction in areas like the cranial nerves, cerebellum, and spine or elevated intracranial pressure [1]. Patients commonly experience headaches, mental status changes, nausea, and vomiting, along with symptoms of radiculopathies, myelopathies, and cauda equina syndrome. Diplopia, facial weakness, and hearing changes may arise due to the involvement of the oculomotor, facial, and cochlear nerves [1]. As these symptoms are non-specific, it is essential to consider other diagnoses, including infectious meningitis, treatment-related effects, and paraneoplastic syndromes.

LMD therefore represents a rare and serious complication of advanced malignancy, particularly in the setting of breast cancer, as it typically reflects a progression to metastatic disease [2]. The incidence of LMD in patients newly diagnosed with intracranial malignant disease has been found to be 11.4% with breast primary, whereas it is just 2.9% in non-breast primaries [3]. Leptomeningeal involvement carries a poor prognosis [2]. However, mortality in breast cancer patients with LMD is relatively lower with a median survival of 5.2 months in contrast to those with non-breast primaries that display higher mortality and poorer prognosis with a median survival of only 2.4 months [3]. Other malignancies associated with LMD include melanoma, non-Hodgkin's lymphoma, and non-small cell cancer of the lung [4]. The complexity of LMD diagnosis stems from its diverse clinical presentations, which can mimic other neurological or infectious conditions, posing challenges to clinicians [2]. The case presented here outlines the rapid deterioration and the diagnostic difficulties encountered in a patient with advanced breast cancer and suspected LMD.

## Case presentation

Presentation

A lady in her mid-40s presented to the emergency department of a district general hospital with a two-week history of headaches. She localized the headache to bi-temporal, retro-orbital, and occipital regions associated with neck stiffness. The headache would start as a severe pain, graded as 10/10, with sudden onset for a few minutes followed by persistent dull ache. It was non-radiating, worsened on lying flat, and improved on sitting up. She reported an increased frequency of these episodes, occurring every 15 minutes, at the time of admission to the hospital. However, there was no vomiting, photophobia, fever, or rash. There was no reported vision loss initially. The headache was accompanied by two episodes of fainting as described by the patient's relatives which were later confirmed as absence seizure episodes. She had been admitted with similar complaints for one week in the hospital and was discharged once the headache got better but re-presented with worsening symptoms within three days. She was hemodynamically stable on admission with a blood pressure (BP) of 120/73, a pulse rate of 66 bpm, a respiratory rate of 18 bpm, a maintaining oxygen saturation of 98% on room air, and a temperature of 36.7°C.

**Figure 1:**
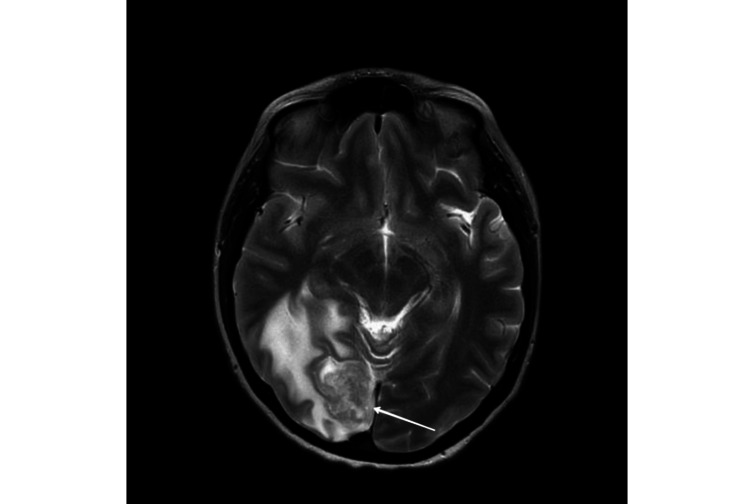
MRI of the head with contrast T2 FLAIR demonstrating occipital metastasis. MRI: magnetic resonance imaging; FLAIR: fluid-attenuated inversion recovery

**Figure 2:**
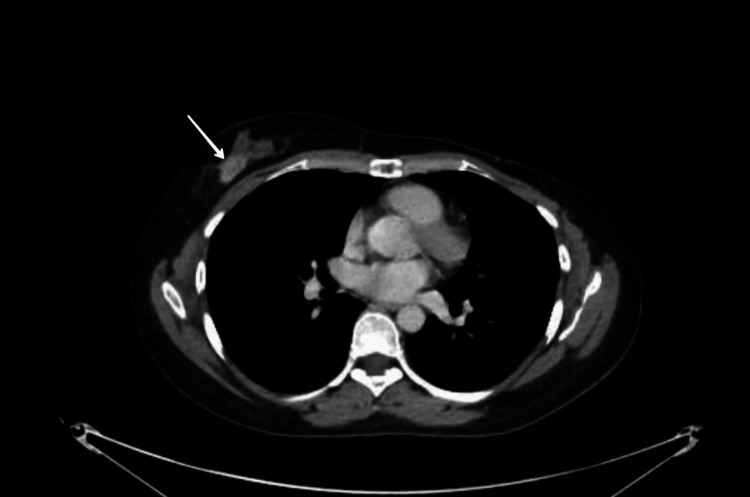
CT of the thorax, abdomen, and pelvis with contrast cross-section showing a new breast malignant lesion. CT: computed tomography

**Figure 3:**
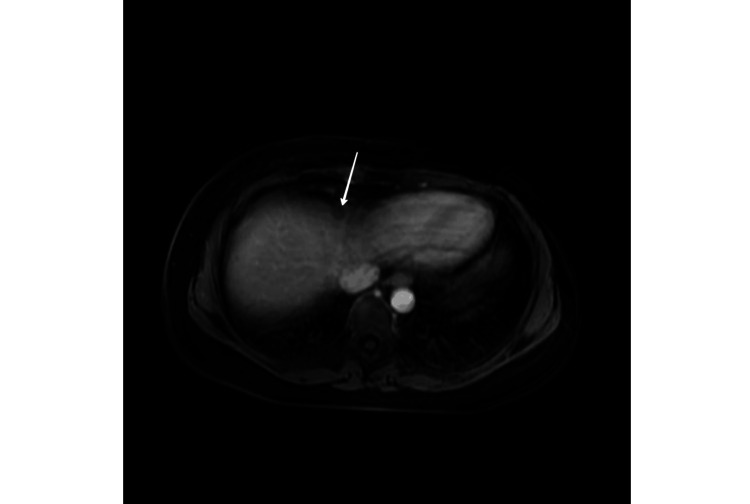
MRI of the liver with contrast T2 image demonstrating a benign hepatic cyst. MRI: magnetic resonance imaging

She had a background of advanced metastatic inflammatory breast cancer. She was diagnosed with grade 3 invasive ductal carcinoma (IDC) in her right breast almost three years ago in pregnancy. At the time of diagnosis, she had T2N1MX stage malignancy with her left breast being triple negative. She received neoadjuvant chemotherapy with paclitaxel-carboplatin and epirubicin-cyclophosphamide followed by left mastectomy and axillary lymph node clearance two years ago. Subsequently, adjuvant radiotherapy was administered to the left chest wall and supraclavicular fossa. She had been offered adjuvant olaparib but she decided against it. A couple of months before presentation, magnetic resonance imaging (MRI) of the head revealed a solitary heterogeneously enhancing lesion in the occipital region in keeping with brain metastasis (Figure 1). Furthermore, a computed tomography (CT) scan of the neck, thorax, abdomen, and pelvis showed a 2.1 × 1.3 cm contrast-enhancing lesion in the right breast raising the suspicion of either a new malignant breast lesion or a metastatic deposit from the previously known left-sided breast cancer (Figure 2) while also showing multiple well-defined liver lesions, which were confirmed to be hepatic cysts without evidence of metastasis on MRI of the liver (Figure 3). She underwent imaging-guided occipital parasagittal craniotomy and near-total resection of metastasis. The histology demonstrated scores of 0 and 2+ in estrogen receptor (ER)/human epidermal growth factor receptor 2 (HER2), BReast CAncer gene 1 (BRCA1) positivity, and D-dish positivity. She also received stereotactic radiosurgery (SRS) to the resected cavity. She was planned for further MRI of the liver and breast, echocardiogram, and positron emission tomography-computed tomography (PET-CT) and was due to commence the first cycle of chemotherapy with docetaxel and pertuzumab, trastuzumab, and hyaluronidase-zzxf (PHESGO); however, this was postponed due to her admission to the hospital with headaches.

During admission, she complained of diplopia, worse on the right lateral gaze, and was found to have partial sixth cranial nerve (abducens) palsy on examination due to failure of abduction of the right eye. Her visual acuity progressively worsened during admission, unable to see her hands. However, on assessment, no ophthalmological abnormality was detected to explain the patient's loss of vision suggesting the likelihood of a neurological cause behind her symptoms.

Furthermore, she started to have episodes of unresponsiveness with up-rolling eyeballs without tonic-clonic jerks, associated with dilated pupils that were sluggish to react and a drop in the Glasgow Coma Scale (GCS) to 3 (E1V1M1). These were assessed to be episodes of absence seizures that were triggered by severe headaches or head movements, for instance, while getting up or sitting up. She had neck stiffness but didn't have photophobia or rashes. She had multiple episodes of absence seizures throughout admission with increasing frequency and duration, requiring intravenous lorazepam to terminate.

Investigations

**Figure 4:**
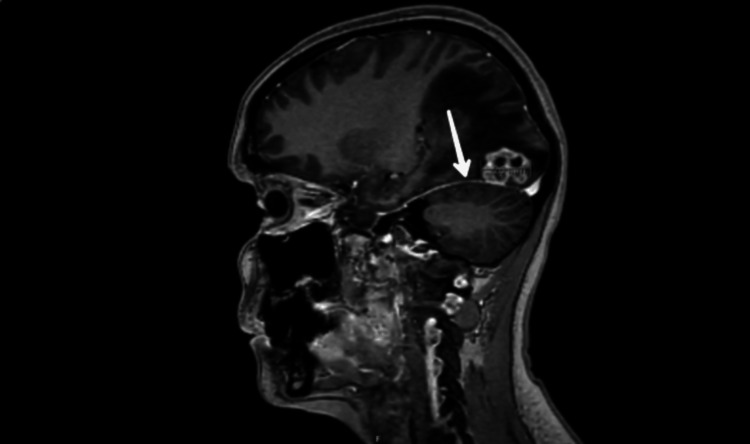
MRI of the head with contrast sagittal section demonstrating evidence of cerebellar surface deposits in keeping with leptomeningeal disease. MRI: magnetic resonance imaging

Blood investigations at hospital presentation were essentially within normal range. She underwent a CT of the head that didn't reveal any acute intracranial abnormalities. Leptomeningeal involvement was suspected keeping her background of metastatic breast cancer in mind. A lumbar puncture was performed that yielded clear, colorless CSF with an opening pressure of 3 cm of water. CSF findings were suggestive of a possibility of meningitis with raised protein, low glucose relative to plasma, and raised CSF white cell count which was predominantly lymphocytic (Table 1). CSF viral polymerase chain reaction (PCR) resulted in negative for varicella zoster virus (VZV), herpes simplex virus (HSV), enterovirus, and parechovirus with no bacterial growth being detected on the CSF culture. The initial CSF cytology specimen was inconclusive for malignancy. She did have some risk factors for tuberculosis (TB) as she was born in Moldova and lived in Russia with a possible history of TB in her grandfather. MRI of the orbits with contrast was performed that showed the optic nerve was tortuous with optic nerve sheath thickening with enhancement suggesting the possibility of raised intracranial pressure. There was no space-occupying lesion identified. Malignancy remained the primary differential with treatment also ongoing for bacterial meningitis, while further investigations were awaited. A repeat MRI of the head with contrast was performed and was also reviewed by a multidisciplinary team involving neuroradiologists. It reported a slight prominence of lateral ventricles and an enhancement within the auditory canal, bilateral optic nerves, and bilateral facial and vestibulocochlear nerves in addition to the coating of the surface of the cerebellum that was concerning for LMD, either neoplastic or infectious (Figure 4). There were no parenchymal change and no evidence of recurrence of metastatic disease at the region of excised metastasis at the right occipital lobe. Electroencephalography (EEG) was performed that showed non-specific findings with no evidence of interictal epileptiform activity. There was mild diffuse slowing of background rhythm which was intermittently asymmetrical being slower in the right hemisphere and intermixed with a moderate amount of low-amplitude diffuse fast activity. A moderate to large amount of medium- to high-amplitude slow wave activities were also seen which were generalized as well as multifocal predominantly in the temporal regions. MRI of the whole spine was requested but could not be successfully done due to patient movement and seizure episode in the radiology department. A repeat lumbar puncture was done primarily aimed at looking at cytology with sufficient sample size. The repeat CSF cytology test was able to demonstrate large atypical mononuclear cells, suspicious for malignant cells.

**Table 1:** Investigation results of CSF cell count, lactate, total protein, and glucose. CSF: cerebrospinal fluid

Investigations	Units	Results	Normal reference range
CSF glucose	mmol/L	1.2	2.2-4.4
CSF total protein	g/L	1.51	0.15-0.45
CSF lactate	mmol/ L	7.6	1.1-2.4
CSF red cell count	×10^6^/L	15	0
CSF white cell count	×10^6^/L	161 (neutrophils 1%, lymphocytes 72%)	0-5

Treatment

The patient was admitted to the hospital under a general medical ward with multidisciplinary input being received from relevant specialties like neurology, oncology, and palliative medicine through consultations. Initial treatment included starting her on dexamethasone 4 mg tablets once daily due to a history of brain metastasis and recent craniotomy for the resection of occipital metastasis. Liquid oxycodone was started to optimize analgesia. Amitriptyline 20 mg was also added. She was later started on oxycodone via a syringe driver as a continuous subcutaneous infusion with the uptitration of the dose to optimize pain relief.

There was no indication of any neurosurgical intervention based on the above scans. She was started on treatment for suspected aseptic meningitis with intravenous ceftriaxone. Due to her having unresponsive episodes, she was put on cardiac monitoring and assessed for critical care admission but didn't fulfill the criteria since she had no organ support needs. Due to recurrent absence seizures, she was given a loading dose of levetiracetam 4 g intravenously and started on a regular dosage which was uptitrated up to 1500 mg twice daily. During episodes of seizures, she frequently required intravenous lorazepam to terminate seizures. Intravenous amoxicillin 2 g twice daily was added to cover for *Listeria monocytogenes* infection given she had raised lymphocytes in her CSF. Antiviral therapy in the form of acyclovir was not initiated as imaging was not consistent with encephalitis and viral CSF PCR had returned negative. Given the risk factors for TB and progressive deterioration despite treatment for bacterial meningitis, in view of diagnostic uncertainty, she was started on treatment for TB meningitis with isoniazid, rifampicin, pyrazinamide, and levofloxacin alongside ceftriaxone and amoxicillin. Consideration was given to wean down dexamethasone but was continued at 8 mg due to concerns regarding meningitis. She continued to have frequent seizure episodes despite being re-loaded with levetiracetam and increased regular dose of 1500 mg twice daily. Hence, sodium valproate 600 mg three times daily was added. Following continuous deterioration over a span of 10 days, she was assessed to be approaching the end of life. Antibiotics were stopped, and priority was given to symptom control with dual anti-epileptics (lorazepam, sodium valproate) with midazolam added, if required, in the event of recurrent seizures. She was deemed to be for ward-level care only and would not be for resuscitation in the event of a cardiac arrest. Dexamethasone dose was increased to 32 mg daily in two divided doses. Clonazepam 1000 mg and midazolam 20 mg were later added to the syringe driver along with oxycodone to attempt control of seizures. Clonazepam was later increased to 1500 mg with oxycodone being uptitrated to 70 mg and midazolam to 50 mg in the syringe driver. CSF TB PCR returned negative following which TB medications were stopped. The results of the repeat MRI of the head and repeat CSF cytology confirmed LMD secondary to metastatic breast cancer. The establishment of malignant pathology implied poor prognosis and guided management towards a more palliative approach. Eventually, due to rapid and continued deterioration, she was declared to be approaching the end of life, and her medications were stopped except for anti-epileptics and anticipatory medications, that is, oxycodone, midazolam, and clonazepam. Unfortunately, the patient eventually succumbed to her illness and passed away a couple of days later within 17 days of presentation to the hospital.

## Discussion

This case highlights several atypical features and challenges that were faced throughout the patient's hospital course. Firstly, the rapid progression of her neurological symptoms, including her bi-temporal and occipital headaches, absence seizures, and eventual loss of vision, was concerning for significant intracranial pathology. Despite multiple imaging modalities, the definitive diagnosis of LMD remained elusive until advanced stages, underscoring the diagnostic difficulties associated with this condition [5].

The atypical presentation in this patient includes the absence of classic signs like photophobia, nausea, or vomiting, which are often associated with raised intracranial pressure or meningitis. Moreover, her history of advanced metastatic inflammatory breast cancer adds complexity to the diagnosis. The coexistence of multiple pathologies, including brain metastases, a potential new malignant lesion in the contralateral breast, and the possibility of leptomeningeal involvement, required a high degree of clinical suspicion and reliance on multidisciplinary inputs [6].

Her neurological decline, characterized by unresponsiveness and frequent seizures, necessitated aggressive medical management, including high doses of antiepileptics and corticosteroids. CSF cytology has been regarded as the gold standard investigation modality with a sensitivity of 50-60% that increases to 80% with repeat collection [4]. The CSF findings, with elevated protein, low glucose, and the presence of large atypical mononuclear cells suspicious for malignancy, were in keeping with LMD, but the rapid worsening of her clinical state posed additional challenges for management. The decision to empirically treat for TB meningitis further complicated her case, though the absence of improvement in TB treatment and subsequent negative PCR results led to the discontinuation of that therapy [7]. Treatment for LMD has undergone development in recent times with cranial irradiation being the choice to de-bulk the cancer load and provide symptomatic relief. Systemic anti-cancer therapy or chemotherapy can also be added to prolong survival [4]. However, none of the above could be offered to the patient due to her rapid deterioration, the challenges encountered to reach a diagnosis, and the repetitive seizure episodes that had been unresponsive to steroids. She was very unwell to receive any anti-cancer treatment, so much so that she died less than three weeks after presentation and within a few days of confirmation of LMD diagnosis.

The progressive nature of this case, particularly with a marked deterioration over just 17 days, highlights the aggressiveness of LMD in this context. The patient's transition from headache and diplopia to a state of refractory seizures and end-of-life care was rapid, and despite best efforts, her condition proved to be intractable [8]. Her death within 17 days of presentation reflects the poor prognosis associated with leptomeningeal metastases, particularly in the context of advanced breast cancer with prior brain involvement [8].

## Conclusions

This case exemplifies the challenges clinicians face in diagnosing and managing complex oncological and neurological conditions with overlapping presentations. The clinical presentation of LMD closely mimics other acute neurological diseases like meningitis and central nervous system (CNS) TB as was observed in this case, thus making an early and accurate diagnosis incredibly difficult. Requesting radiological investigations, with MRI of the head with contrast being the investigation of choice, is a reasonable approach; however, findings on the scan can be very subtle and often require scan images to be reviewed by specialist neuroradiologists to highlight any suspicious radiological finding. A good knowledge of malignancies known to cause LMD is essential. This lady presented with severe headache and deterioration of visual acuity with seizure activity in the form of absence seizures on a background of advanced metastatic breast cancer. Breast cancer is one of the commonest malignancies associated with LMD, and even though the clinicians rightly suspected the possibility of LMD in light of the known breast malignancy, reaching a diagnosis posed considerable challenges given the non-specific presentation, CSF results reflective of a possibility of CNS infection, and complex interpretation of MRI changes. Importantly, LMD, in the context of malignancy, carries a poor prognosis. Hence, an early diagnosis is imperative in guiding management towards a palliative approach. Early suspicion, a thorough investigation, and a multidisciplinary approach remain crucial in managing such patients, though the rapid progression of this case serves as a stark reminder of the aggressive nature of LMD and its associated poor outcomes.
